# Novel extensions of *k*-harmonically convex functions and their applications in information science

**DOI:** 10.1371/journal.pone.0320192

**Published:** 2025-07-01

**Authors:** Asfand Fahad, Shigeru Furuichi, Zammad Ali, Yuanheng Wang

**Affiliations:** 1 School of Software, Pingdingshan University, Pingdingshan, China; 2 Henan International Joint Laboratory for Multidimensional Topology and Carcinogenic Characteristics Analysis of Atmospheric Particulate Matter PM2.5, Pingdingshan, China; 3 Centre for Advanced Studies in Pure and Applied Mathematics, Bahauddin Zakariya University, Pakistan; 4 Department of Information Science, College of Humanities and Sciences, Nihon University, Setagaya-ku, Tokyo, Japan; 5 Department of Mathematics, Saveetha School of Engineering, SIMATS, Thandalam, Chennai, Tamilnadu, India; 6 School of Mathematical Sciences, Zhejiang Normal University, Jinhua, China; PLOS, UNITED STATES OF AMERICA

## Abstract

Convex analysis theory has found extensive applications in optimization, information science, and economics, leading to numerous generalizations of convex functions. However, a drawback in the vast literature on convex functions is that only a limited number of these notions significantly impact practical applications. With this context, we explore a novel convexity notion known as *k*-harmonically convex function (*k*-HCF) using two approaches and present applications in information science. First, we propose an *r*-parameterized extension of *k*-HCF, broadening its applicability. Secondly, we extend this concept to interval-valued functions (IVFs), based on a complete order relation on closed bounded intervals. We then investigate properties and inequalities for both extensions to derive lower bounds for information-theoretic measures such as Tsallis entropy, Shannon entropy, and Tsallis relative entropy, using the new parametric extensions of these functions. Additionally, we prove inequalities of the Jensen, Mercer, and Hermite-Hadamard types for the Cr-order-based extension of *k*-HCFs. Our findings reproduce known results while introducing significant new insights into the field, showing the broader usefulness of *k*-HCFs in information science.

## 1 Introduction

Convex analysis theory has drawn considerable interest from scholars due to its extensive applications in optimization, information science, economics, and other diverse fields, see [1–3]. This discipline has experienced numerous enhancements and developments over the years. The properties of convex functions (CFs), such as continuity, differentiability, and monotonicity of derivatives, distinguish them as a critical mathematical tool utilized across various disciplines. As a result, different variants of CFs have been introduced and explored, leading to the discovery of both classical and novel inequalities. For more comprehensive information on certain derived classes of CFs and corresponding study, see [4] and the references therein. Among the other extensions of the CFs, a significant one is the class of harmonic convex functions (HCFs), which has been introduced [5] and studied extensively. This class has been studied in context of inequalities and entropy [6–8], generalizations and extensions [9, 10]. The family of HCFs and its modified/extended versions have strong connections with mathematical inequalities, for example, HH-type inequalities for multiplicatively HCFs have been established in [11]. One recent generalization of HCFs with applications in Physics, analysis and information sciences, known as *k*-harmonically convex functions, has been given in [12]. Recently, the extension of CFs for interval-valued functions (IVFs) has remained the topic of interest, see []. A significant step towards interval-valued convex functions (IVCFs) is the introduction of complete order defined on the compact intervals in real line is the *Cr* order relation. The *Cr*-order enables the extension of variants of real-valued CFsto IVFs, known as GA-*Cr*-CFs [13]. Moreover, inequalities of Hadamard and Mercer types along with the applications of *Cr*-order based CFs in information sciences have also been presented in [13–15].

On the other hand, mathematical inequalities is among the most studied topics in mathematics. It is because of their vast applicability in studying key components of modern and classical phenomena. In recent years, mathematical inequalities are applied to investigate system reliability prediction, feedback control and stability analysis [16, 17]. Specifically, fundamental inequalities involving Jensen’s, Gr$\ddot{o}$n-wall’s, H$\ddot{o}$lder’s and Bihari’s present direct utilities towards the investigations of differential equations controllability [18], Chebyshev’s inequality is applied to determine neighborhood boundaries of data points [19]. All this result in to investigations of mathematical inequalities in multiple object domains. Therefore, in recent years, variants of several other inequalities including Jensen’s and Jensen-Mercer (JI and JMI, respectively) and Hermite-Hadamard inequality (HHI) [20, 21], Heinz-type inequalities for convex functions [22], Young and Polya-scego-type inequalities [23] have been proved via novel and classical approaches. HH-type inequalities for interval-valued *h*-preinvex functions via fractional integral operators (FIOs) has been proved in [24]. Among the formal study of mathematical inequalities, JI, JMI and HHI have dominated the subject. In [25], HHI via Yang’s fractal theory, parameterized local fractional inequalities for generalized *h*-preinvex functions with applications in numerical integration have been proved. In [26], fractional HHI and fractional Bullen inequality via stochastic orderings techniques have been proved. The HHI for the β-integral and the well known quantum integrals has been proved in [27]. A strong connection between information theory with JI and JMI via entropy has been presented in [28]. Linear inequalities play a crucial role in determining the dynamics, solution conditions, and long-term behaviors in anti-symmetric Lotka-Volterra systems, with significance in linear programming theory. In addition, various applications based on fractional-order models can be found in [29, 30], and numerous inequalities are also derived using fractional derivatives and integrals.

The Shannon entropy [31] and the Tsallis entropy [32] are among the most studied notions in information theory, pivotal for advancements in understanding diverse probabilistic and statistical models. Compared to Shannon entropy, generalized entropy and parametric optimization have shown better sensitivity to rotor dynamics [33], highlighting the need to study the properties of generalized entropies. The *Cr*-order based parametric extensions of *Cr*-CFs has produced generalization of extended log-sum inequality [34], the notion of *Cr*-relative entropy [13] which reproduces the *f*-divergence [35] (see also [36]), Tsallis relative entropy [37], Kullback–Leibler information [38] and possess the properties including non–negativity, monotonicity and joint convexity [37].

Due to importance of *k*-HCFs, parametric extensions of CFs and the *Cr*-order based extensions of CFs, we introduce two extensions of *k*-HCFs. Firstly, we extend the notion of *k*-HCF with a parameter *r*. Secondly, we extend this concept to interval-valued functions (IVFs), based on a complete order relation on closed bounded intervals. We then investigate properties and inequalities for both extensions to derive lower bounds for information-theoretic measures such as Tsallis entropy [32], Shannon entropy [31], and Tsallis relative entropy [37], using the new parametric extensions of these functions. Additionally, we prove inequalities of the Jensen, Mercer, and Hermite-Hadamard types for the Cr-order-based extension of *k*-HCFs and recaptures well-known results from [5, 8, 12, 39, 40]. As a result, we reproduce known results and cover both theoretical aspect (in convex analysis and inequalities) and application aspect (in information sciences) in our study.

Before presenting the main findings of the manuscript, we include necessary notions for the ease of the readers. For notions related to the Riemann integral and its properties, addition and the scalar multiplication of the intervals, *Cr*-order relation, *Cr*-CFs we refer [13] to the readers. We further use ℜ, ${ℜ}^{+}$, ${ℜ}_{I}$ and ${ℜ}_{I}^{+}$, to denote real numbers, positive real numbers, compact intervals in ℜ and the positive compact intervals in ${ℜ}^{+}$, respectively. Now, we include the notion of novel *k*-HCF:

**Definition 1.1.** [12] *A function φ:[μ,ν]→ℜ is said to be a k-HCF, where k∉[μ,ν], if*

$$\varphi \left(\frac{1}{\frac{\tau }{x-k}+\frac{1-\tau }{y-k}}+k\right)\leq \tau \varphi (x)+(1-\tau )\varphi (y),$$ (1)

*holds for any x,y∈[μ,ν] and 0≤τ≤1.*

Before we show our results, we review the recently proved inequalities from [12] and mention related inequalities from the recent literature.

**Theorem 1.2** (Jensen Type inequality). *([12; Theorem 2.3]) For [μ,ν] in ℜ, k∉[μ,ν] and a k-HCF φ:[μ,ν]→ℜ, the inequality*


$$\varphi \left(\frac{1}{\sum_{j=1}^{n}\frac{p_{j}}{x_{i}-k}}+k\right)\leq \sum_{i=1}^{n}p_{i}\varphi (x_{i})$$

*holds for all $x_{i}\in [\mu ,\nu ]$, and $p_{i}\geq 0$ with $\sum_{i=1}^{n}p_{i}=1$.*

**Theorem 1.3.** *[12, Theorem 2.9]) [HH-Type Inequality] For a k-HCF φ on [μ,ν], the inequalities*


$$\varphi \left(\frac{2}{\frac{1}{\nu -k}+\frac{1}{\mu -k}}+k\right)\leq \frac{\left(\nu -k)(\mu -k\right)}{\nu -\mu }\int_{\mu }^{\nu }\frac{\varphi (x)dx}{(x-k{)}^{2}}\leq \frac{\varphi (\mu )+\varphi (\nu )}{2}$$

*hold.*

**Theorem 1.4.** *[12, Theorem 2.11]) [JM-Type Inequality] For a k-HCF φ on [μ,ν], the inequality*


$$\varphi \left(\frac{1}{\frac{1}{\nu -k}+\frac{1}{\mu -k}-\sum_{i=1}^{n}\frac{p_{i}}{x_{i}-k}}+k\right)\leq \varphi (\mu )+\varphi (\nu )-\sum_{i=1}^{n}p_{i}\varphi (x_{i})$$

*hold for all $x_{i}\in [\mu ,\nu ]$, and $p_{i}\geq 0$ with $\sum_{i=1}^{n}p_{i}=1$.*

## Main results

The main results of the manuscript have been presented in the following two sections.

## 2 Parametric extensions of *k*-harmonically convex functions and information theoretic measures

In the current section, we prove results to obtain parametric extensions of *k*-HCFs. Further, we present applications of the new extensions by obtaining new lower bounds for several well-known information-theoretic measures such as Tsallis entropy, Shannon entropy, and Tsallis relative entropy.

To obtain a parametric extension of [12; Example 3.3], we use *r*–logarithmic function $\ln_{r}(x):=\frac{x^{r}-1}{r},\;\;(x>0,\;\;r\neq 0)$, which uniformly converges to the usual logarithmic function logx as r→0.

To prove Proposition 2.2, we prepare the following lemma.

**Lemma 2.1.** *Assume 0≤μ≤x<y≤ν and k∉[μ,ν]. Let t∈[0,1] and φ be real-valued twice differentiable function on [μ,ν]. If $\frac{d^{2}}{dt^{2}}\varphi \left(\frac{1}{\frac{t}{x-k}+\frac{1-t}{y-k}}+k\right)\geq 0$, then φ is k-HCF on [μ,ν].*

*Proof*: We set the function


$$g(t):=t\varphi (x)+(1-t)\varphi (y)-\varphi \left(\frac{1}{\frac{t}{x-k}+\frac{1-t}{y-k}}+k\right),\;\;(0\leq t\leq 1).$$

Since $\frac{d^{2}g(t)}{dt^{2}}=-\frac{d^{2}\varphi \left(\frac{1}{\frac{t}{x-k}+\frac{1-t}{y-k}}+k\right)}{dt^{2}}\leq 0$ by the assumption, we have g(t)≥0 from the fact g(0)=g(1)=0. ◻

**Proposition 2.2.** *For 0≤μ<ν and a real number k:*

(i) *If k>ν and −1≤r≤1 with r≠0, then $-\ln_{r}(x)$ is k-HCF on [μ,ν].*
(ii) *If k≤−ν and *r*<0, then $-\ln_{r}(x)$ is k-HCF on [μ,ν].*

*Proof*: Let *x* and *y* be such that 0≤μ≤x<y≤ν. We define a function h:[0,1]→ℜ by


$$h(t):=\frac{1}{r}\left(1-{\left(\frac{1}{\frac{t}{x-k}+\frac{1-t}{y-k}}+k\right)}^{r}\right).$$

Then, we have


$$\frac{d^{2}h(t)}{dt^{2}}=\frac{\left(k-x)(k-y)(x-y{)}^{2}{\left(\frac{1}{\frac{t}{x-k}+\frac{1-t}{y-k}}+k\right)}^{r}g(t\right)}{{\left(k-(1-t)x-ty\right)}^{2}{\left(xy-k\left(tx+(1-t)y\right)\right)}^{2}},$$

where


$$g(t):=k^{2}(1-r)-(1+r)xy+k\left((2t-1+r)x+(1+r-2t)y\right).$$

Since $\frac{dg(t)}{dt}=2k(x-y)<0$ for k>ν, the minimum value of *g*(*t*) is non–negative:


$$g(1)=(1-r)k^{2}+\left((1+r)x-(1-r)y\right)k-(1+r)xy=(k-y)\left((1-r)k+(1+r)x\right)\geq 0$$

by −1≤r≤1 and *k*>*y* (from k>ν). Therefore we have $\frac{d^{2}h(t)}{dt^{2}}\geq 0$ so that (i) was proven by Lemma 2.1.

For the case (ii) :k≤−ν, we have $\frac{dg(t)}{dt}=2k(x-y)\geq 0$. Then the minimum value of *g*(*t*) is positive:


$$g(0)=(1-r)k^{2}+k\left((r-1)x+(r+1)y\right)-(1+r)xy=(k-x)\left((1-r)k+(1+r)y\right)\;\geq (1-r)(-y)(k-x)+(1+r)y(k-x)=2ry(k-x)>0$$

by *r*<0 and k≤−y (from k≤−ν). Therefore we have $\frac{d^{2}h(t)}{dt^{2}}\geq 0$ so that (ii) was also proven by Lemma 2.1. ◻

Letting r→0 in Proposition 2.2, we find that −logx is a *k*-HCF on [μ,ν] whenever k≤−ν or k>ν. These conditions satisfy k∉[μ,ν] in [12, Definition 2.2].

Since $-\ln_{r}x\neq \ln_{r}\frac{1}{x},\;\;(x>0,\;\;r\neq 0)$ in general, we prove the following.

**Proposition 2.3.** *For 0≤μ<ν and a real number k:*

(i) *If k>ν and −1≤r≤1 with r≠0, then $\ln_{r}(1/x)$ is a k-HCF on [μ,ν].*
(ii) *If k≤−ν and *r*>0, then $\ln_{r}(1/x)$ is a k-HCF on [μ,ν].*

*Proof*: Let *x* and *y* be such that 0≤μ≤x<y≤ν. We define a function h:[0,1]→ℜ by


$$h(t):=\frac{1}{r}\left({\left(\frac{1}{\frac{t}{x-k}+\frac{1-t}{y-k}}+k\right)}^{-r}-1\right).$$

Then, we have


$$\frac{d^{2}h(t)}{dt^{2}}=\frac{\left(k-x)(k-y)(x-y{)}^{2}{\left(\frac{1}{\frac{t}{x-k}+\frac{1-t}{y-k}}+k\right)}^{-r}g(t\right)}{{\left(k-(1-t)x-ty\right)}^{2}{\left(xy-k(tx+(1-t)y)\right)}^{2}}$$

where


$$g(t):=k^{2}(1+r)-(1-r)xy-k\left((r+1-2t)x+(r-1+2t)y\right).$$

Since $\frac{dg(t)}{dt}=2k(x-y)<0$ for k>ν, the minimum value of *g*(*t*) is non–negative:


$$g(1)=(1+r)k^{2}+\left((1-r)x-(1+r)y\right)k-(1-r)xy=(k-y)\left((1+r)k+(1-r)x\right)\geq 0$$

by −1≤r≤1 and *k*>*y* (from k>ν). Therefore we have $\frac{d^{2}h(t)}{dt^{2}}\geq 0$ so that (i) was proven by Lemma 2.1.

For the case (ii): k≤−ν, we have $\frac{dg(t)}{dt}=2k(x-y)>0$. Then the minimum value of *g*(*t*) is positive for *r*>0 and k≤−y (from k≤−ν):


$$g(0)=(1+r)k^{2}-\left((1+r)x-(1-r)y\right)k-(1-r)xy=(k-x)\left((1+r)k+(1-r)y\right)>0.$$

Indeed, from k≤−ν we have k≤−y. We also have $-y<\left(\frac{r-1}{1+r}\right)y$ for *r*>0 and *y*>0. Thus we have $k\leq -y<\left(\frac{r-1}{1+r}\right)y$ so that (1+r)k+(1−r)y<0. Therefore we have $\frac{d^{2}h(t)}{dt^{2}}\geq 0$ so that (ii) was also proven by Lemma 2.1. ◻

Letting r→0 in Proposition 2.3, we find that −logx is *k*-HCF on [μ,ν] when k≤−ν or k>ν. These conditions satisfy k∉[μ,ν] as in [12, Definition 2.2].

**Remark 2.4.** *We give a short remark on Proposition 2.2 and 2.3. The function $-\ln_{r}(x),(x>0,\;\;r\neq 0)$ is convex on x∈(0,∞) when r≤1. The function $\ln_{r}\left(1/x\right),\;\;(x>0,\;\;r\neq 0)$ is also convex on x∈(0,∞) when r≥−1. Therefore both functions $-\ln_{r}(x)$ and $\ln_{r}\left(1/x\right)$ are convex on x∈(0,∞) when −1≤r≤1 with r≠0. We thus find that the results obtained in Proposition 2.2 and 2.3 are quite natural.*

For a probability distribution $p:=\{p_{1},\cdots ,p_{n}\}$ and r≠0, the Tsallis entropy [32] was defined by


$$T_{r}\left(p\right):=\sum_{j=1}^{n}p_{j}\ln_{r}\frac{1}{p_{j}}=-\sum_{j=1}^{n}p_{j}^{1-r}\ln_{r}p_{j}=\sum_{j=1}^{n}\left(\frac{p_{j}^{1-r}-p_{j}}{r}\right).$$

It is known [32, 37] that $0\leq T_{r}\left(p\right)\leq \ln_{r}n$. The non–negativity of $T_{r}\left(p\right)$ is shown by monotone decreasingness of $\ln_{r}\frac{1}{x}$. The maximality of $T_{r}\left(p\right)$ is shown by the non–negativity of the Tsallis relative entropy, see [39, Remark 1.3]. Applying Proposition 2.3 with Jensen type inequality shown in [12, Theorem 2.3], we give a lower bound of the Tsallis entropy $T_{r}\left(p\right)$.

**Theorem 2.5.** *Let $p:=\{p_{1},\cdots ,p_{n}\}$ be a probability distribution and let $p_{\max}:=\max_{1\leq j\leq n}\{p_{j}\}$ and $p_{\min}:=\min_{1\leq j\leq n}\{p_{j}\}$. If −1≤r≤1 with r≠0 and $k>p_{\max}$, then*

$$0\leq \ln_{r}\frac{1}{p_{\max}}\leq \ln_{r}{\left(\frac{1}{\sum_{j=1}^{n}\frac{p_{j}}{p_{j}-k}}+k\right)}^{-1}\leq T_{r}\left(p\right).$$ (2)

*If r* > 0 *and* $k\leq -p_{\max}$, *then we have the inequalities* (2).

*Proof*: From Proposition 2.3, the function $\ln_{r}\frac{1}{x}$ is *k*-HCF on $\left[p_{\min},p_{\max}\right]$ when −1≤r≤1 with r≠0 and $k>p_{\max}$, where we set $x_{j}:=p_{j}$ in Theorem 1.2. Applying Theorem 1.2, we have


$$\ln_{r}{\left(\frac{1}{\sum_{j=1}^{n}\frac{p_{j}}{p_{j}-k}}+k\right)}^{-1}\leq \sum_{j=1}^{n}p_{j}\ln_{r}\frac{1}{p_{j}}.$$

Since $p_{j}\;-\;k\leq p_{\max}\;-\;k<0$, we have $\sum_{j=1}^{n}\frac{p_{j}}{p_{j}-k}\geq \sum_{j=1}^{n}\frac{p_{j}}{p_{\max}-k}=\frac{1}{p_{\max}-k}$ which implies $\frac{1}{\sum_{j=1}^{n}\frac{p_{j}}{p_{j}-k}}\;+\;k\leq \frac{1}{\frac{1}{p_{\max}-k}}\;+\;k=p_{\max}\leq 1$. Thus we have the desired inequality (2), since $\ln_{r}\frac{1}{x},\;\;(x>0)$ is a monotone decreasing function w.r.t. *x* for all r≠0 and $\ln_{r}1=0$.

Taking $x_{j}:=p_{j}$ for all j=1,⋯,n and $\phi (x):=\ln_{r}\frac{1}{x}$ in Theorem 1.2, we have the second inequality in (2). Since $p_{\max}$ − $k\geq p_{j}$ − *k*>0 (from k≤ − $p_{\max}$), we have $\sum_{j=1}^{n}\frac{p_{j}}{p_{j}-k}\geq \sum_{j=1}^{n}\frac{p_{j}}{p_{\max}-k}=\frac{1}{p_{\max}-k}$. Thus we have $\frac{1}{\sum_{j=1}^{n}\frac{p_{j}}{p_{j}-k}}+k\leq \frac{1}{\frac{1}{p_{\max}-k}}+k=p_{\max}\leq 1$, which implies the first and the second inequalities in (2) since $\ln_{r}\frac{1}{x},\;\;(x>0)$ is a monotone decreasing function w.r.t. *x* for all r≠0 and $\ln_{r}1=0$. ◻

It is remarkable that we have for $k>p_{\max}$ or $k\leq -p_{\max}$,


$$0\leq -\log p_{\max}\leq -\ln_{r}\left(\frac{1}{\sum_{j=1}^{n}\frac{p_{j}}{p_{j}-k}}+k\right)\leq S(p)$$

where $S(p):=-\sum_{j=1}^{n}p_{j}\log p_{j}$ is the Shannon entropy [31], by letting r→0 in Theorem 2.5.

The Tsallis relative entropy [37] is defined by


$$D_{r}\left(p|q\right):=-\sum_{j=1}^{n}p_{j}\ln_{r}\frac{q_{j}}{p_{j}}=\sum_{j=1}^{n}\left(\frac{p_{j}-p_{j}^{1-r}q_{j}^{r}}{r}\right)$$

for two probability distributions $p:=\{p_{1},\cdots ,p_{n}\}$ and $q:=\{q_{,}\cdots ,q_{n}\}$ with parameter r≠0.

**Theorem 2.6** *Let* $p:=\{p_{1},\cdots ,p_{n}\}\;\mathrm{and}\;q:=\{q_{1},\cdots ,q_{n}\}$ *be probability distribution and let* $(q/p{)}_{\max}:=\max_{1\leq j\leq n}\left\{\frac{q_{j}}{p_{j}}\right\}$ *and* $(q/p{)}_{\min}:=\min_{1\leq j\leq n}\left\{\frac{q_{j}}{p_{j}}\right\}$, *where we assume p*_*j*_>0 *for all* j=1,⋯,n. If −1≤r≤1 *with* r≠0 *and* $k>(q/p{)}_{\max}$, *then*

$$-\ln_{r}{\left(q/p\right)}_{\max}\leq -\ln_{r}\left(\frac{1}{\sum_{j=1}^{n}\frac{p_{j}}{q_{j}/p_{j}-k}}+k\right)\leq D_{r}\left(p|q\right)$$ (3)

If *r*<0 and $k\leq -(q/p{)}_{\max}$, then we have the inequalities (3).

*Proof*: From Proposition 2.2, $-\ln_{r}x$ is *k*–HCF on $\left[(q/p{)}_{\min},(q/p{)}_{\max}\right]$. Applying Theorem 1.2 with $x_{j}:=\frac{q_{j}}{p_{j}}$, we have the last inequality in (3) for both cases:

(i) −1≤r≤1 with r≠0 and $k>(q/p{)}_{\max}$,
(ii) *r*<0 and $k\leq -(q/p{)}_{\max}$.

For the case (i), we have $0>(q/p{)}_{\max}$ − $k\geq \frac{q_{j}}{p_{j}}$ − $k\;\mathrm{which}\;\mathrm{implies}\;0>\sum_{j=1}^{n}\frac{p_{j}}{q_{j}/p_{j}-k}\geq \sum_{j=1}^{n}\frac{p_{j}}{(q/p{)}_{\max}-k}=\frac{1}{(q/p{)}_{\max}-k}$. Thus we have

$$\frac{1}{\sum_{j=1}^{n}\frac{p_{j}}{q_{j}/p_{j}-k}}+k\leq \frac{1}{\frac{1}{(q/p{)}_{\max}-k}}+k=(q/p{)}_{\max}$$ (4)

Since $-\ln_{r}x,\;\;(x>0)$ is monotone decreasing w.r.t. xfor allr≠0, we have the first inequality in (3).

For the case (ii), we have $0<\frac{q_{j}}{p_{j}}$ − $k\leq (q/p{)}_{\max}$ − $k\;\mathrm{which}\;\mathrm{implies}\;0<\sum_{j=1}^{n}\frac{p_{j}}{(q/p{)}_{\max}-k}\leq \sum_{j=1}^{n}\frac{p_{j}}{q_{j}/p_{j}-k}$. Thus we have the inequality (4). Thus we also have the first inequality in (3). ◻

We should note that $-\ln_{r}{\left(q/p\right)}_{\max}\leq 0$, since ${\left(q/p\right)}_{\max}\geq 1$. Otherwise, there exsits two probability distributions p and q such that $q_{j}<p_{j}$ for all j=1,⋯,n. This contradicts the condition $\sum_{j=1}^{n}p_{j}=1=\sum_{j=1}^{n}q_{j}$. Therefore, the inequality $-\ln_{r}{\left(q/p\right)}_{\max}\leq D_{r}\left(p|q\right)$ does not give an improvement of the non—-negativity of the Tsallis relative entropy such as $D_{r}\left(p|q\right)\geq 0$.

## 3 Interval-valued k-Harmonically-Cr-convex functions and Inequalities

We first introduce the notion of the *Cr*−*k*– Harmonically convex function (Crk-HCF).

**Definition 3.1** *A function $F:J\to {ℜ}_{I}$, denoted as $F=[\underset{―}{F},\bar{F}]$ is said to be a Cr−k–Harmonically convex function on *J* if the inequality*

$$F\left(\frac{1}{\frac{\tau }{\rho -k}+\frac{1-\tau }{\xi -k}}+k\right){≼}_{cr}\tau F(\rho )+(1-\tau )F(\xi ),$$ (5)

*holds for any 0≤τ≤1,ρ,ξ∈J and k,0∉J.*

**Remark 3.2.**

1. *If $\underset{―}{F}(s)=\bar{F}(s),s\in J$ in Definition 3.1 then we get k-HCF as in [12].*
2. *If we take *k* = 0 in Definition 3.1 then, we get Cr-harmonically convex function (Cr-HCF) as in [39]*
3. *If take $\underset{―}{F}(s)=\bar{F}(s),s\in J\;\mathrm{and}\;k=0$ in Definition 3.1 then we get harmonically-convex function (HCF) as in [5].*

**Proposition 3.3.** *Let $F:J\subseteq ℜ⧵\{k\}\to {ℜ}_{I}^{+}$ be an IVF. If $F_{c}\;and\;F_{r}\;are\;k-\mathrm{HCFs}\;on\;J\;\mathrm{then}\;F\;is\;Cr-k-HCF\;on\;J$*.

*Proof*: Given that $F_{c}\;\mathrm{and}\;F_{r}\;\mathrm{are}\;k-\mathrm{HCFs}\;\mathrm{on}\;J$, then for given *k*, for any ρ,ξ∈J and τ∈(0,1) the inequalities


$$F_{c}\left(\frac{1}{\frac{\tau }{\rho -k}+\frac{1-\tau }{\xi -k}}+k\right)\leq \tau F_{c}(\rho )+(1-\tau )F_{c}(\xi )$$

and


$$F_{r}\left(\frac{1}{\frac{\tau }{\rho -k}+\frac{1-\tau }{\xi -k}}+k\right)\leq \tau F_{r}(\rho )+(1-\tau )F_{r}(\xi )$$

hold. Now, if $F_{c}\left(\frac{1}{\frac{\tau }{\rho -k}+\frac{1-\tau }{\xi -k}}+k\right)\neq \tau F_{c}(\rho )+(1-\tau )F_{c}(\xi )\;\mathrm{for}\;\mathrm{each}\;\tau \in (0,1)\;\mathrm{and}\;\rho ,\xi \in J$ then


$$F_{c}\left(\frac{1}{\frac{\tau }{\rho -k}+\frac{1-\tau }{\xi -k}}+k\right)<\tau F_{c}(\rho )+(1-\tau )F_{c}(\xi ),$$

therefore


$$F\left(\frac{1}{\frac{\tau }{\rho -k}+\frac{1-\tau }{\xi -k}}+k\right){≼}_{cr}\tau F(\rho )+(1-\tau )F(\xi ).$$

Otherwise, if $F_{c}\left(\frac{1}{\frac{\tau }{\rho -k}+\frac{1-\tau }{\xi -k}}+k\right)=\tau F_{c}(\rho )+(1-\tau )F_{c}(\xi )\;\mathrm{for}\;\mathrm{some}\;\tau \in (0,1)\;\mathrm{or}\;\rho ,\xi \in J$, even then for every τ∈(0,1) and ρ,ξ∈J,


$$F_{r}\left(\frac{1}{\frac{\tau }{\rho -k}+\frac{1-\tau }{\xi -k}}+k\right)\leq \tau F_{r}(\rho )+(1-\tau )F_{r}(\xi ),$$

which yields


$$F\left(\frac{1}{\frac{\tau }{\rho -k}+\frac{1-\tau }{\xi -k}}+k\right){≼}_{cr}\tau F(\rho )+(1-\tau )F(\xi )$$

to prove that F is Cr−k-HCF. ◻

In the next result, we formulate and prove the JI for the *Cr*−*k*-HCFs.

**Theorem 3.4** (Jensen’s inequality for *Cr*−*k*-HCFs). *For a Cr−k−HCF on J, and for any $\rho_{i}\in J$, $0\leq \tau_{i}\leq 1\;\mathrm{with}\;\sum_{i=1}^{n}\tau_{i}=1$ the inequality*

$$F\left(\frac{1}{\sum_{j=1}^{n}\frac{\tau_{j}}{\rho_{j}-k}}+k\right){≼}_{cr}\sum_{j=1}^{n}\tau_{j}F(\rho_{j})$$ (6)

*holds.*

*Proof*: To prove the inequality, we apply induction on *n*. The case when *n* = 2 is evident from Definition 3.1. Now, assume that (6) holds under the assumption of Theorem 3.4 for *n* − 1. To prove (6) for the case *n*, consider


$$F\left(\frac{1}{\sum_{j=1}^{n}\frac{\tau_{j}}{\rho_{j}-k}}+k\right)=F\left(\frac{1}{\sum_{j=1}^{n-2}\frac{\tau_{j}}{\rho_{j}-k}+\frac{\tau_{n-1}}{\rho_{n-1}-k}+\frac{\tau_{n}}{\rho_{n}-k}}+k\right)=\;F\left(\frac{1}{\sum_{j=1}^{n-2}\frac{\tau_{j}}{\rho_{j}-k}+\left(\frac{\left(\tau_{n-1}+\tau_{n}\right)}{k+(\tau_{n-1}+\tau_{n})\left\{\frac{\left(\rho_{n-1}-k)(\rho_{n}-k\right)}{\tau_{n-1}(\rho_{n}-k)+\tau_{n}(\rho_{n-1}-k)}\right\}-k}\right)}+k\right)\;{≼}_{cr}\sum_{j=1}^{n-2}\tau_{j}F(\rho_{j})+(\tau_{n-1}+\tau_{n})\;\;\;\;\;\;\;\;\;\;\;F\left(k+(\tau_{n-1}+\tau_{n})\left\{\frac{\left(\rho_{n-1}-k)(\rho_{n}-k\right)}{\tau_{n-1}(\rho_{n}-k)+\tau_{n}(\rho_{n-1}-k)}\right\}\right)\;\;\;\;\;(by\;\;\;induction\;\;\;hypothesis)\;=\sum_{j=1}^{n}\tau_{j}F(\rho_{j})+(\tau_{n-1}+\tau_{n})F\left(\frac{1}{\frac{1}{\tau_{n-1}+\tau_{n}}\frac{\tau_{n-1}}{\rho_{n-1}-k}+\frac{1}{\tau_{n-1}+\tau_{n}}\frac{\tau_{n}}{\rho_{n}-k}}+k\right)\;\;\;\;\;{≼}_{cr}\sum_{j=1}^{n-2}\tau_{j}F(\rho_{j})+(\tau_{n-1}+\tau_{n})\left\{\frac{\tau_{n-1}}{\tau_{n-1}+\tau_{n}}F(\rho_{n-1})+\frac{\tau_{n}}{\tau_{n-1}+\tau_{n}}F(\rho_{n})\right\}(by\;\;\;Definition\;3.1)\;=\sum_{j=1}^{n}\tau_{j}F(\rho_{j}),$$

which completes the proof. ◻

**Remark 3.5.**

1. *If we take $\underset{―}{F}(s)=\bar{F}(s),s\in J$ in Theorem 3.4, then we obtain Theorem 2.3 proved in [12].*
2. *Further, if we take $\underset{―}{F}(s)=\bar{F}(s),s\in J\;\mathrm{and}\;k=0$ in Theorem 3.4, then we obtain the Theorem 4.3 proved in [40].*

By taking $\tau_{i}=\frac{1}{n}$ in Theorem 3.4, we obtain the following result.

**Corollary 3.6.** *For a $Cr-k-\mathrm{HCF}\;on\;J\;with\;k\notin J\;and\;for\;any\;\rho_{j}\in J$, the inequality*


$$F\left(\frac{n}{\sum_{j=1}^{n}\frac{\tau_{j}}{\rho_{j}-k}}+k\right){≼}_{cr}\frac{\sum_{j=1}^{n}\tau_{j}F(\rho_{j})}{n}$$

*holds.*

Before proving the JMI for *Cr* − *k*-HCFs, we prove an important property related to order for subtraction of the intervals. The property is particularly important because the cancellation property does not hold for this interval operations.

**Proposition 3.7.** *Consider $A=[\underset{―}{a},\bar{a}]$, $B=[\underset{―}{b},\bar{b}]\;\mathrm{and}\;C=[\underset{―}{c},\bar{c}]$. If $A+B{≼}_{cr}C\;\mathrm{then}\;A{≼}_{cr}C-B$.*

*Proof*: Under the given assumption, it is sufficient to establish the following inequalities:


$$A_{c}\leq (C-B{)}_{c}$$

and


$$A_{r}\leq (C-B{)}_{r}.$$

Since $(A+B{)}_{c}\leq C_{c}\;\mathrm{therefore}\;\bar{a}+\underset{―}{a}+\bar{b}+\underset{―}{b}\leq \bar{c}+\underset{―}{c}\;\mathrm{implies}\;A_{c}=\frac{\bar{a}+\underset{―}{a}}{2}\leq \frac{\bar{c}-\underset{―}{b}+\underset{―}{c}-\bar{b}}{2}=(C-B{)}_{c}$. For the second inequality, since $(A+B{)}_{r}\leq C_{r}\;\mathrm{therefore}\;\bar{a}-\underset{―}{a}+\bar{b}-\underset{―}{b}\leq \bar{c}-\underset{―}{c}$ which implies


$$A_{r}=\frac{\bar{a}-\underset{―}{a}}{2}\leq \frac{\left(\bar{c}-\underset{―}{c})+(\underset{―}{b}-\bar{b}\right)}{2}\leq \frac{\left(\bar{c}-\underset{―}{c})+(\bar{b}-\underset{―}{b}\right)}{2}=(C-B{)}_{r},$$

which was required. ◻

**Lemma 3.8.** *For a *Cr* − *k* − $\mathrm{HCF}\;\mathrm{on}\;[\mu ,\nu ]\;\mathrm{with}\;k\notin [\mu ,\nu ]\;\mathrm{and}\;for\;any\;\rho_{j}\in [\mu ,\nu ]$, the inequality*


$$F\left(\frac{1}{\frac{1}{\mu -k}+\frac{1}{\nu -k}-\frac{1}{\rho -k}}+k\right){≼}_{cr}F(\mu )+F(\nu )-F(\rho )$$

*holds.*

*Proof*: Let μ≤ρ≤ν, there exist τ∈[0,1] such that


$$\frac{1}{\rho -k}=\frac{\tau }{\mu -k}+\frac{1-\tau }{\nu -k}.$$

By applying the Definition 3.1, we get

$$F(\rho )=F\left(\frac{1}{\frac{\tau }{\mu -k}+\frac{1-\tau }{\nu -k}}+k\right){≼}_{cr}\tau F(\mu )+(1-\tau )F(\nu ).$$ (7)

On the other side

$$F\left(\frac{1}{\frac{1}{\mu -k}+\frac{1}{\nu -k}-\frac{1}{\rho -k}}+k\right)=F\left(\frac{1}{\frac{1-\tau }{\mu -k}+\frac{\tau }{\nu -k}}+k\right){≼}_{cr}(1-\tau )F(\mu )+(\tau )F(\nu ).$$ (8)

By adding (7) and (8) along with the Proposition 3.7, we get


$$F\left(\frac{1}{\frac{1}{\mu -k}+\frac{1}{\nu -k}-\frac{1}{\rho -k}}+k\right){≼}_{cr}F(\mu )+F(\nu )-F(\rho ),$$

which was required. ◻

Now, we are ready to prove the JMI for *Cr*−*k*-HCFs.

**Theorem 3.9.** *For a Cr−k−HCF on [μ,ν] with k∉[μ,ν], the inequality*

$$F\left(\frac{1}{\frac{1}{\mu -k}+\frac{1}{\nu -k}-\sum_{j=1}^{n}\frac{\tau_{j}}{\rho_{j}-k}}+k\right){≼}_{cr}F(\mu )+F(\nu )-\sum_{j=1}^{n}\tau_{j}F(\rho_{j})$$ (9)

*holds for all $\mu \leq \rho_{j}\leq \nu$, $\tau_{j}\geq 0\;\mathrm{with}\;1\leq j\leq n\;\mathrm{satisfying}\;\sum_{j=1}^{n}\tau_{j}=1$.*

*Proof*: From Theorem 3.4, we get

$$F\left(\frac{1}{\frac{1}{\mu -k}+\frac{1}{\nu -k}-\sum_{j=1}^{n}\frac{\tau_{j}}{\rho_{j}-k}}+k\right)=F\left(\frac{1}{\sum_{j=1}^{n}\tau_{j}\left(\frac{1}{\mu -k}+\frac{1}{\nu -k}-\frac{1}{\rho_{j}-k}\right)}+k\right){≼}_{cr}\;\sum_{j=1}^{n}\tau_{j}F\left(\frac{1}{\frac{1}{\mu -k}+\frac{1}{\nu -k}-\frac{1}{\rho_{j}-k}}+k\right).$$ (10)

Further, from the Lemma 3.8, we obtain

$$\sum_{j=1}^{n}\tau_{j}F\left(\frac{1}{\frac{1}{\mu -k}+\frac{1}{\nu -k}-\frac{1}{\rho_{j}-k}}+k\right){≼}_{cr}F(\mu )+F(\nu )-\sum_{j=1}^{n}\tau_{j}F(\rho_{j}).$$ (11)

By utilizing (11) in (10), we achieve the desired result. ◻

**Remark 3.10.** *If take $\underset{―}{F}(s)=\bar{F}(s),s\in J$ in Theorem 3.9 then we obtain Theorem 2.11 from [12]. Additional assumption k=0 produces Theorem2.4 from [8].*

Now, we prove the HHI for *Cr*−*k*-HCF.

**Theorem 3.11.** *For a Cr−k−HCF on [μ,ν] with k∉[μ,ν], the inequalities*

$$F\left(\frac{2}{\frac{1}{\mu -k}+\frac{1}{\nu -k}}+k\right){≼}_{cr}\frac{\left(\mu -k)(\nu -k\right)}{\nu -\mu }\int_{\mu }^{\nu }\frac{F(s)}{(s-k{)}^{2}}ds{≼}_{cr}\frac{F(\mu )+F(\nu )}{2},$$ (12)

*hold.*

*Proof*: The second inequality follows directly by taking ρ=μ, ξ=ν in (5) and integrating w.r.t. τ from 0 to 1. For the first inequality, consider (5) again and take $\tau =\frac{1}{2}$, then we have

$$F\left(\frac{2}{\frac{1}{\rho -k}+\frac{1}{\xi -k}}+k\right){≼}_{cr}\frac{F(\rho )+F(\xi )}{2},$$ (13)

for all ρ,ξ∈[μ,ν].

By substituting, $\rho =\frac{1}{\frac{\tau }{\mu -k}+\frac{1-\tau }{\nu -k}}+k\;\mathrm{and}\;\xi =\frac{1}{\frac{1-\tau }{\mu -k}+\frac{\tau }{\nu -k}}+k$ in (13), we have

$$F\left(\frac{2}{\frac{1}{\rho -k}+\frac{1}{\xi -k}}+k\right){≼}_{cr}\frac{F\left(\frac{1}{\frac{\tau }{\mu -k}+\frac{1-\tau }{\nu -k}}+k\right)+F\left(\frac{1}{\frac{1-\tau }{\mu -k}+\frac{\tau }{\nu -k}}+k\right)}{2}.$$ (14)

The rest follows from integrating both sides w.r.t. τ from 0 to 1. ◻

**Remark 3.12.** *The special case $\underset{―}{F}(s)=\bar{F}(s),s\in [\mu ,\nu ]$ of Theorem 3.11 yields Theorem 2.9 from [12]. In addition, the case when k=0 coincides with the Theorem 2.4 from [5]. On the other hand, if we assume *k* = 0 in Theorem 3.11 then we get the HHI for Cr−HCFs as given in Remark 3 of [41].*

**Theorem 3.13.** *For a Cr−k−HCF on [μ,ν] with k∉[μ,ν], the inequalities*

$$F\left(\frac{2}{\frac{2}{\nu -k}+\frac{2}{\mu -k}-\frac{1}{\rho -k}-\frac{1}{\xi -k}}+k\right){≼}_{cr}\frac{\left(\rho -k)(\xi -k\right)}{\xi -\rho }\int_{\rho }^{\xi }\frac{F(z)}{(z-k{)}^{2}}dz\;{≼}_{cr}F(\mu )+F(\nu )-\frac{F(\rho )+F(\xi )}{2}$$ (15)

*hold for all μ≤ρ≠ξ≤ν.*

*Proof*: Let 0≤τ≤1 and applying Theorem 3.11 to get:

$$F\left(\frac{2}{\frac{2}{\nu -k}+\frac{2}{\mu -k}-\frac{1}{\rho -k}-\frac{1}{\xi -k}}+k\right)\;\;{≼}_{cr}\frac{1}{2}F\left(\frac{1}{\tau \left(\frac{1}{\nu -k}+\frac{1}{\mu -k}-\frac{1}{\rho -k}\right)+(1-\tau )\left(\frac{1}{\nu -k}+\frac{1}{\mu -k}-\frac{1}{\xi -k}\right)}+k\right)\;\;{≼}_{cr}\frac{1}{2}F\left(\frac{1}{(1-\tau )\left(\frac{1}{\nu -k}+\frac{1}{\mu -k}-\frac{1}{\rho -k}\right)+\tau \left(\frac{1}{\nu -k}+\frac{1}{\mu -k}-\frac{1}{\xi -k}\right)}+k\right).$$ (16)

Since


$$\int_{0}^{1}\bar{F}\left(\frac{1}{\tau \left(\frac{1}{\nu -k}+\frac{1}{\mu -k}-\frac{1}{\rho -k}\right)+(1-\tau )\left(\frac{1}{\nu -k}+\frac{1}{\mu -k}-\frac{1}{\xi -k}\right)}+k\right)d\tau \;\;=\int_{0}^{1}\bar{F}\left(\frac{1}{\tau \left(\frac{1}{\nu -k}+\frac{1}{\mu -k}-\frac{1}{\rho -k}\right)+(1-\tau )\left(\frac{1}{\nu -k}+\frac{1}{\mu -k}-\frac{1}{\xi -k}\right)}+k\right)d\tau \;\;=\frac{\left(\rho -k)(\xi -k\right)}{\xi -\rho }\int_{\rho }^{\xi }\frac{1}{(u-k{)}^{2}}\bar{F}\left(\frac{1}{\nu -k}+\frac{1}{\mu -k}-\frac{1}{u-k}\right)du\;\;=\frac{\left(\rho -k)(\xi -k\right)}{\xi -\rho }\int_{\rho }^{\xi }\frac{\bar{F}(z)}{(z-k{)}^{2}}dz$$

and similarly


$$\int_{0}^{1}\underset{―}{F}\left(\frac{1}{\tau \left(\frac{1}{\nu -k}+\frac{1}{\mu -k}-\frac{1}{\rho -k}\right)+(1-\tau )\left(\frac{1}{\nu -k}+\frac{1}{\mu -k}-\frac{1}{\xi -k}\right)}+k\right)d\tau \;\;=\frac{\left(\rho -k)(\xi -k\right)}{\xi -\rho }\int_{\rho }^{\xi }\frac{\underset{―}{F}(z)}{(z-k{)}^{2}}dz.$$

Therefore, the component wise definition of the integral of IVFs implies

$$\int_{0}^{1}F\left(\frac{1}{\tau \left(\frac{1}{\nu -k}+\frac{1}{\mu -k}-\frac{1}{\rho -k}\right)+(1-\tau )\left(\frac{1}{\nu -k}+\frac{1}{\mu -k}-\frac{1}{\xi -k}\right)}+k\right)d\tau \;\;=\frac{\left(\rho -k)(\xi -k\right)}{\xi -\rho }\int_{\rho }^{\xi }\frac{F(z)}{(z-k{)}^{2}}dz$$ (17)

Similarly,

$$\int_{0}^{1}F\left(\frac{1}{(1-\tau )\left(\frac{1}{\nu -k}+\frac{1}{\mu -k}-\frac{1}{\rho -k}\right)+\tau \left(\frac{1}{\nu -k}+\frac{1}{\mu -k}-\frac{1}{\xi -k}\right)}+k\right)d\tau \;\;=\frac{\left(\rho -k)(\xi -k\right)}{\xi -\rho }\int_{\rho }^{\xi }\frac{F(z)}{(z-k{)}^{2}}dz.$$ (18)

Further, integrate (3) w.r.t. τ from 0 to 1 and utilizing (17) and (18) we obtain the first inequality of (15). For the second inequality in (15), we apply Theorem 3.9 as:


$$F\left(\frac{1}{\frac{1}{\nu -k}+\frac{1}{\mu -k}-\frac{\tau }{\rho -k}-\frac{1-\tau }{\xi -k}}+k\right){≼}_{cr}F(\mu )+F(\nu )-\tau F(\rho )-(1-\tau )F(\xi ).$$

Further, integrate the above inequality w.r.t. τ from 0 to1, which yields the second inequality in (15).

**Corollary 3.14.** *For a Cr−k−HCF on [μ,ν] with k∉[μ,ν]:*

1. *if μ≤ρ≤ν, then*
  $$F\left(\frac{1}{\frac{1}{\nu -k}+\frac{1}{\mu -k}-\frac{1}{\rho -k}}+k\right){≼}_{cr}F(\rho ){≼}_{cr}\frac{F(\mu )+F(\nu )}{2}.$$ (19)
2. *The following inequalities hold*
$$\frac{1}{\mu -\nu }\int_{\mu }^{\nu }F\left(\frac{1}{\frac{1}{\mu -k}+\frac{1}{\nu -k}+\frac{1}{\rho -k}}+k\right)d\rho {≼}_{cr}\frac{1}{\mu -\nu }\int_{\mu }^{\nu }F\left(\rho \right)d\rho {≼}_{cr}\frac{F(\mu )+F(\nu )}{2}.$$

*Proof*:

1. *From (15), the desired inequality is obtained as ξ→ρ.*
2. We obtain the required inequality by integrating both sides of (19) with respect to ρ over [μ,ν].

◻

**Remark 3.15.** *Under the special case when $\underset{―}{F}(s)=\bar{F}(s),s\in [\mu ,\nu ]$, the inequalities from the Theorem 3.13 and the Corollary 3.14 yield Theorem 2.12 and the Corollary2.13 from [12].*

## 4 Examples

In this section, we establish some examples from the proved results and analyze the impact of the parameter *k* and the IVFs based inequalities. We also comment on the improvement of HH-Type inequalities for particular examples.

**Example 1.** *Consider the function* $F(s)=[\underset{―}{F}(s),\bar{F}(s)]=[s^{2},s^{2}]\;\mathrm{for}\;s\in [1,2]$. *By the Example* 3.2(1) *of [12], both the functions* $\underset{―}{F}\;and\;\bar{F}\;are\;k\;-\;\mathrm{HCFs}\;on\;[1,2]$. *Consequently, F is Cr-k-HCF on* [1, 2] *and by utilizing* (12), *we obtain:*


$${\left(\frac{4-3k}{3-2k}\right)}^{2}\leq (1-k)(2-k)\int_{1}^{2}\frac{s^{2}}{(s-k{)}^{2}}\;ds\leq 2.5.$$

**Fig 1:**
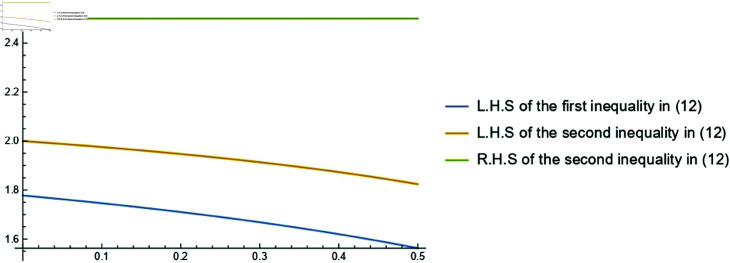
Graphical representation of (12) $\mathrm{for}\;s^{2}\;\mathrm{for}\;\mathrm{the}\;\mathrm{parameter}\;0\leq k<\frac{1}{2}$.

*Under the assumptions, it is evident that the second inequality is optimal for *k* = 0. The graphical representation of the assumed case for $0\leq k<\frac{1}{2}$ is shown in* Fig 1.

**Example 2.** *Consider the function $F(s)=[\underset{―}{F}(s),\bar{F}(s)]=[-\frac{log(s)}{s},-\frac{log(s)}{s}]\;\mathrm{for}\;s\in [e,e^{\frac{3}{2}}]$. By the Example 3.6 of [12], both the functions $\underset{―}{F}\;and\;\bar{F}\;are\;\mathrm{Cr-k-HCFs}\;on\;[e,e^{\frac{3}{2}}]$. Consequently, $F\;\mathrm{is}\;\mathrm{Cr-k-HCF}\;on\;[e,e^{\frac{3}{2}}]\;\mathrm{and}\;by\;utilizing$* (12), *we obtain:*


$$-\frac{\log\left(\frac{2(e-k)(e^{3/2}-k)}{e^{3/2}+e-2k}+k\right)}{\frac{2(e-k)(e^{3/2}-k)}{e^{3/2}+e-2k}+k}\leq -\frac{\left(e-k)(e^{3/2}-k\right)}{e\left(e^{1/2}-1\right)}\int_{e}^{e^{3/2}}\frac{\log(s)}{s(s-k{)}^{2}}\;ds\leq -\frac{1}{2}\left(\frac{1}{e}+\frac{3/2}{e^{3/2}}\right).$$

**Fig 2:**
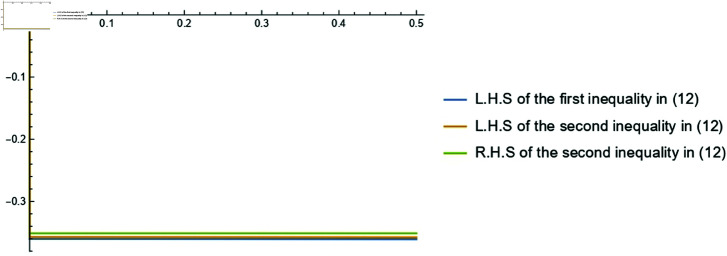
Graphical representation of (12) $\mathrm{for}-\frac{log(s)}{s}\;\mathrm{for}\;\mathrm{the}\;\mathrm{parameter}\;0\leq k<\frac{1}{2}$.

*The graphical representation of the assumed case for $0\leq k<\frac{1}{2}$ is shown in* Fig 2.

**Example 3.** *Consider the function $F(s)=[\underset{―}{F}(s),\bar{F}(s)]=[0,2s^{2}]\;for\;s\in [1,2]$. Then, both $F_{c}=F_{r}=s^{2}\;are\;k\;-\;\mathrm{HCFs}\;on\;[1,2]$. Consequently, by the Proposition 3.3, F is Cr-k-HCF on [1,2] and by utilizing* (12), *we obtain:*


$$\left[0,2{\left(\frac{4-3k}{3-2k}\right)}^{2}\right]{≼}_{cr}\left[0,(1-k)(2-k)\int_{1}^{2}\frac{2s^{2}}{(s-k{)}^{2}}\;ds\right]{≼}_{cr}[0,5].$$

**Fig 3:**
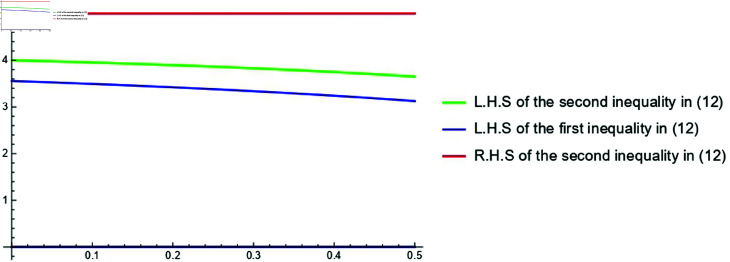
Graphical representation of ( 12) for [0,2s^2^] for the parameter $0\leq k<\frac{1}{2}$.

*The graphical representation of the assumed case for $0\leq k<\frac{1}{2}$ is shown in Fig 3.*

## Conclusion

In this article, we have introduced and explored novel extensions of *k*-HCFs and demonstrated their significant applications in the field of information sciences. By extending the notion of the *k*-HCF with two approaches (i) via a parameter *r* and (ii) for the IVFs, we have broadened the scope and applicability of convex functions. More precisely, we have produced new properties and inequalities for both extensions: providing valuable lower bounds for information-theoretic measures such as Tsallis entropy, Shannon entropy, and Tsallis relative entropy. The derivation of inequalities of the Jensen, Mercer, and Hermite-Hadamard types for the *Cr*-order based extension of *k*-HCFs has been particularly noteworthy, as it not only reproduces known results but also extends them, thus enriching the theoretical framework of convex analysis. More specifically, our study has recaptured main results from [5, 8, 12, 41, 42]. In conclusion, our extensions in the family of CFs have provided both theoretical and application advancements in the subject. It would be interesting to investigate the impact of the newly introduced functions through *k*-HCFs and fractional integrals, and to extend the studies presented in [43, 44] and subsequent research.
